# Validation of Affect-tag Affective and Cognitive Indicators

**DOI:** 10.3389/fninf.2021.535542

**Published:** 2021-05-10

**Authors:** Laurent Sparrow, Hugo Six, Lauren Varona, Olivier Janin

**Affiliations:** ^1^Univ. Lille, CNRS, CHU Lille, UMR 9193—SCALab—Sciences Cognitives et Sciences Affectives, Lille, France; ^2^Neotrope, Tourcoing, France

**Keywords:** affective computing, cognitive load, wearable, bio-signal, electrodermal acitivity, emotional states discrimination

## Abstract

The Affect-tag solution measures physiological signals to deliver indicators derived from cognitive science. To provide the most accurate and effective results, a database of electrodermal activity (EDA) signals acquired using the Affect-tag A1 band was created. An experimental paradigm was designed to measure action-taking, autonomic regulation, cognitive load (CL), emotions, and stress, affects, and social stress. The Affect-tag emotional power (EP), emotional density (ED), and CL affective and cognitive indicators were refined based on the physiological responses of 48 participants during these tasks. Statistical significance was obtained for all indicators in tasks they were designed to measure, resulting in a total accuracy score of 89% for the combined indicators. Data obtained during this study will be further analyzed to define emotional and affective states.

## Introduction

NEOTROPE is a design and creation laboratory made up of hardware, software, and cognitive engineers dedicated to providing scientifically based products to the global market. Specializing in the design, development, integration, and publishing of electronic biometric measurement and software solutions for the processing of physiological data, NEOTROPE makes available intra-and interpersonal affective and cognitive indicators based on neuroscientific and cognitive principles for affective researchers in various fields.

Using principles of embodied cognitive and emotional functioning that can be objectively measured, NEOTROPE has created the Affect-tag solution. Where questionnaires and self-reporting on emotional and cognitive behaviors are subjective and are often difficult to interpret, Affect-tag captures the spontaneous corporal changes that cannot be detected by the naked eye. These minute physiological changes reveal information about mental functions, affective states, and arousal. The relationship between physiological readings and mental functioning is well known and has been used for many years in academic research. Thanks to miniaturization and progress in signal processing algorithms, it is now possible to measure these mobile indicators with only a connected wristband device.

Affect-tag includes a wristband measuring heart rate (HR) and electrodermal activity (EDA) as well as a computerized solution for real-time calculation of cognitive and affective indicators. This article presents an experiment allowing to validate, in a first step, the electrodermal indicators thanks to different tasks affecting differently the physiological responses. So, we want to create a database of individual physiological responses in standardized cognitive and affective tasks. To carry out this sampling and to ensure that it complies as closely as possible with current scientific standards, NEOTROPE collaborated with the teams of the SCALab laboratory (Centre National de la Recherche Scientifique and the University of Lille) for their methodological expertise, recruitment, and procurement of samples, and rigorous criteria ofmeasurements.

## Theoretical Framework

### Interpreting Physiological Signals

The body’s electrodermal and cardiac responses can be transformed into indicators that provide information about the functioning of the peripheral nervous system (PNS). The acting arm of the Central Nervous System, the PNS [which includes the somatic and autonomic nervous systems (ANSs)] is able to control the functioning of the peripheral organs and ensure the internal balance of the body independently of any conscious control.

Consequently, HR and EDA are under the influence of both central commands (cognitive and/or conscious) and purely autonomous homeostatic processes (unconscious and triggered by subcortical centers). Among these, the limbic system acts as an interface between cognition and the autonomic division of the PNS: it controls many emotional behaviors, like defense behaviors (stress), aggressiveness, fear, and pleasure. The limbic system is directly linked to the sympathetic branch of the ANS.

In order to distinguish between the different branches and possible origins of the measured signal, a number of precautions must be taken when interpreting them. The most common solution used to control variability is to intentionally induce specific peripheral reactions and sample the signal immediately after stimulation. By comparing the responses obtained during this time window with those collected during other phases (such as those without any induced stimulation), it is possible to evaluate the effects of the stimulation on peripheral responses.

### Physiological Response to Stimuli

The ANS manages the physiology of the body by balancing internal physical and chemical conditions (homeostasis). In the event of a provocation, the body’s resources are mobilized resulting in observable physiological changes. Therefore, if an individual is stimulated or stressed, a momentary physiological imbalance and return to balance will be observed (allostasis, McEwen et al., 2015) and tools exist to measure these direct changes in EDA and HR specifically.

A stressor representing danger or threat is accompanied by a particular configuration of brain electrical activity. This autonomic defensive reaction, commonly known as the fight or flight response, has a significant impact on the body (Cannon, 1987) which can be simulated in order to extract patterns of EDA and HR.

In humans, cardiovascular reactions to mental tasks generally result in increased blood pressure, faster HR, renal vasoconstriction, skeletal muscle vasodilation, and decreased (or steady) vascular resistance (Callister et al., 1992). These responses are caused by an activation of the sympathetic branch of the ANS (Heidbreder et al., 1982). Since this branch also controls the activity of the sweat glands, it is therefore not surprising that mental activities also cause electrodermal reactions (Boucsein, 2012).

The EDA can be separated into its phasic and tonic components that yield information about specific processes. The phasic component, or Skin Conductance Response (SCR), measures rapid transient changes and is related to cognitive and emotional processes and arousal. The tonic component, or Skin Conductance Level (SCL), describes the background activity of the ANS and measures general activation and changes in cognitive processes (Dawson et al., 2007).

Although it is possible to objectively measure cognitive and emotional reactions using these physiological indicators, the majority of scientific studies do not distinguish between the different functions involved in the tasks used or do not systematically study several physiological responses simultaneously in the same individual. This study was therefore carried out in order to allow cardiac and electrodermal measurements to be made by a single measuring device, the Affect-tag A1 band, and for the same individual confronted with different standardized tasks involving cognitive and affective functions.

### Measuring Cognition and Affect

Cognition is the set of mental processes that involve memory, language, reasoning, learning, intelligence, problem-solving, decision making, perception, and attention. All these processes enrich knowledge. The intensity with which these different functions are implemented can be measured and is referred to as cognitive load (CL). The study of cognition is carried out using various scientifically validated and standardized experimental tasks.

The definition of affective functions is much more complex. Emotions are commonly referred to as emotions when they represent only a small part of the various emotional behaviors. Given the complexity of the field, it is necessary to rely on solid and recognized scientific foundations to implement this concept, such as the work of Scherer (2005), which is precise in defining the various concepts. According to him, there are five types of affect: emotions (anger, sadness, joy…), moods (being cheerful, dark, depressed…), interpersonal stances (being distant, warm, caring…), attitudes (sympathetic, affectionate, hateful…), and affect dispositions (nervous, anxious, thoughtless…). These different affects are complex to manipulate and control: emotions are intense and difficult to reproduce experimentally, while moods, postures and attitudes are personality traits that are stable over time but dependent on individuals and therefore difficult to control. On the other hand, affect dispositions are more accessible because they reflect tendencies to experience certain moods and types of emotions, even when the stimulation is low (Scherer, 2005). This definition opens the door to experimental and controlled manipulations because it is possible to create transient (sad, happy…) and moderate moods to objectively measure cognitive and affective functions using different experimental tasks. The results will produce distinct impacts on the physiological functioning of the body that can be measured and interpreted using electrodermal and cardiac indicators.

As mentioned above, these peripheral responses may come from a central control (cognitive or affective) but also from subcortical or autonomic commands. It is therefore necessary to add a task that specifically activates the ANS without the intervention of cognition or other central commands.

## Design of Experimental Procedure

As mentioned above, researchers at the SCALab laboratory designed and conducted a study to collect physiological data using standard tasks from a representative sample of the population. These data, recorded using the Affect-tag band, were used to correlate specific configurations of physiological responses (electrodermal and cardiac activity) with specific psychological states induced by controlled experimental situations.

The psychological states studied are as follows:

• Decision-making and action
• CL:

° Engagement of cognition in a task requiring large mental resources and involving memory, concentration, and mental calculation

• Affective reactions:

° Emotional: situations with positive and negative valence, reactivity to unpleasant memories
° Social: surprise reactions to a situation of social pressure

In addition to these main states, a provoked reflex reaction was also measured through breathing exercises to study autonomous physiological responses.

### Decision Making and Reactivity

To measure cognitive engagement, decision making, and reaction, the participant was prepared (engaged) to detect the appearance of a stimulus and then react by pressing a key on the keyboard. Responses to acute stress are characterized by a sympathetic excitation (arousal) associated with a reciprocal decrease of the parasympathetic activity (vagal withdrawal) but moderate psychological stress, such as that caused by a simple reaction time task, does not imply a reciprocal decrease of the parasympathetic activity (Berntson et al., 1994; McEwen and Wingfield, 2003). The slope of tonic activation of EDA (i.e., electrodermal response rise time) is an indicator of the ability to mobilize cognitive resources (Turpin and Siddle, 1979; Boucsein, 2012). Behavioral response (reaction time) is also a relevant indicator (Powell et al., 1980; Bagherli et al., 2011) regarding EDA interpretation (Wilson, 1987).

### Cognitive Load

Al-Shargie et al. (2016) showed that a mental calculation task based on addition and subtraction using three digits (i.e., 7–3 + 1) from the Montreal Imaging Stress Task (Montreal Imaging Stress Task, Dedovic et al., 2005) provided typical hormonal, physiological and electroencephalographic responses to cognitive stress, particularly when the participant is under time pressure (Al-Shargie et al., 2016). For this task, participants performed 25 increasingly difficult arithmetic operations and wrote the result on a sheet of paper. Compared to the previous task, there is a similar motor component but with a much higher working memory load. In addition to noticeable and progressive sympathetic activation, inhibition of the parasympathetic system should also be observed, particularly on frequency indicators of cardiac variability. The accuracy of the responses can also be correlated with physiological indicators.

### Emotional Tasks

#### Emotional Stress

A video showing the harmful consequences of dangerous and irresponsible driving has the ability to provoke emotional reactions in a systematic way. Sympathetic activation, especially in the phasic component of EDA and the low-frequency HRV, and a pronounced inhibition of the parasympathetic system is expected (Kreibig, 2010). Because of the immersive nature of videos in general, compared to static images, we expect physiological responses that are close to emotional stress. Since events with a strong emotional charge are better retained, three images presenting the faces of the actors in the video are presented at the end of the affective test. This allows testing the effect of the video on the felt emotions: if the participant experiences an emotion, the probability of obtaining physiological responses for these three faces will then be higher.

#### Affective Dispositions

Many studies have examined the effects of emotional stress on peripheral responses (see Kreibig, 2010, for a review). Different types of paradigms have been implemented (presentation of videos, standardized images, sounds, etc.) but the effects of these paradigms on physiological responses are quite modest and variable from one individual to another. However, under certain conditions, the responses can be amplified. For example, when emotional inducers are images, EDA responses are more pronounced for large stimuli (Codispoti and De Cesarei, 2007) and when presented for more than 6 s (Codispoti et al., 2001). Since EDA is also correlated with certain personality variables and anxiety can influence both SCL and SCR (Naveteur and Baque, 1987), the level of anxiety of each participant must be assessed. This was done using the Positive and Negative Affect Schedule (PANAS,Watson et al., 1988) questionnaire.

#### Social Stress

In their article, Kirschbaum et al. (1993) defined a form of public speaking as a trigger for social stress (Trier Social Stress Test, TSST). This situation causes significant hormonal release (ACTH, prolactin, and cortisol) and physiological changes (increase in HR) and are therefore comparable to emotional stress. It also provokes specific responses to phasic characteristics of EDA (mainly amplitude, but also frequency). However, the TSST requires conditions that are complicated to implement (presence of a jury of three people and audio equipment) and requires that the participants be standing and active, which causes motor activation that interferes with purely emotional responses. We used a test similar to the TSST and Sing-a-Song-Stress Test (SSST, Brouwer and Hogervorst, 2014) that is simpler to implement and gives rise to the intended emotional response. In the modified SSST used in this experiment, the participant was asked to sing a song in the presence of the investigator. There was a countdown timer (5 s) for the activity during which social stress increases. However, as the counter reaches zero, the participants were told they did not have to sing after all. This situation is expected to cause sympathetic activation coupled with marked inhibition of the parasympathetic system.

#### Independent Tasks

Breathing exercises included asking the participant to rapidly inhale and slowly exhale. This pattern of breathing leads to parasympathetic activation and decrease sympathetic activation, as observed on EDA measurements (Cappo and Holmes, 1984). This breathing cycle specifically activates the parasympathetic system (Hirsch and Bishop, 1981; Moser et al., 1994; Strauss–Blasche et al., 2000) because respiratory sinus arrhythmia (RSA) is influenced by breathing and is under the exclusive control of the parasympathetic system.

## The Affect-tag Solution

Wristbands are available for measuring physiological parameters but some of them can exhibit some limitations like an external light sensibility (Ollander et al., 2016). Neotrope designed the Affect-tag Band A1 to overcome this kind of limitation, for example, the shape of the band was made to minimize the external light impact on the HR sensor and to optimize the contact between the skin and the EDA sensors. Additionally, Neotrope designed their own wristband so they could control the signal processing chain which is pretty opaque in the case of other wristbands.

After 5 years of research and development, NEOTROPE has created the complete Affect-tag system, a mobile solution for measuring affective and cognitive reactions using physiological data allowing scientists as well as non-scientists to benefit from the complete study of the physiological response of the measurement to the results. Every component of the solution has been designed and developed by the team: the Band A1 to capture physiological data, the signal processing algorithms to extract information, mobile applications to synchronize context, and a cloud architecture for visualization of automatically processed results.

### Hardware and Raw Signal Validation

The Affect-tag A1 band, worn on the wrist like a watch, continuously records EDA, HR, and Acceleration (ACC). Hardware components are medical-grade sensors and materials, including 4 mm Ag/Cl electrodes for precise EDA measurement and a silicon encasing for comfort and portability. The sampling rates are 20 Hz and 50 Hz for EDA and HR, respectively. The band went through a series of in-house and external comparative tests to validate the physiological data acquisition precision using laboratory-grade equipment. The wristband is connected to the Affect-tag mobile application via Bluetooth and transmits filtered EDA and HR which is displayed on the smart device in real-time.

#### Electrodermal Activity

One device for measuring EDA is the BIOPAC Systems, Inc. Values for this device are precise and respond to quick changes due to sensors placed on fingertips and the addition of a gel to amplify the sensitivity of the measurements. The downside of this device is that it is cumbersome, is not mobile, and is expensive. A preliminary test was conducted with SCALab to verify the raw EDA data capture between BioPac and Affect-tag using a series of repeatable protocols to control for expected outcomes.

**Figure 1:**
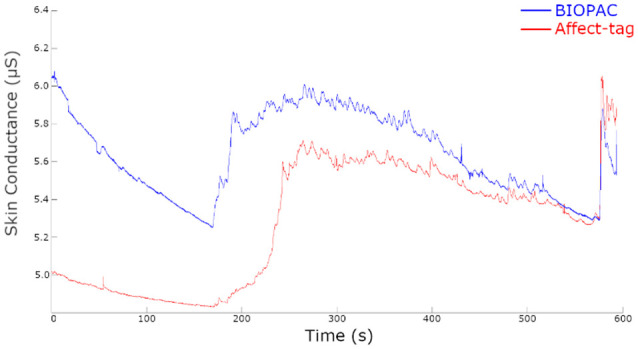
Raw EDA signals for a single participant simultaneously measured using BIOPAC and Affect-tag.

Visual inspection of the raw data acquisition shows comparable results between Affect-tag and BIOPAC (see Figure 1). The lower values registered with Affect-tag are expected since the use of a conducting gel is not used.

Correlation results, obtained from a panel of 10 participants, from the comparison tests yielded the following results (Table 1).

**Table 1:** Average correlation between Affect-tag and BIOPAC signals during specific tasks.

	Correlation Affect-tag—BIOPAC
Cognitive stress	0.87
Social stress	0.92
Rest	0.94
Overall	0.92

Based on these results we concluded that Affect-tag is suitable as a tool for measuring raw Electrodermal responses.

#### Affective and Cognitive Indicators

Neotrope has developed five affective and cognitive indicators to provide clear, straightforward results that represent the emotional experience. These indicators have been created and refined using data obtained in various experimental situations, including laboratory settings (e.g., product testing) and real-world settings (e.g., driving a car).

The indicators are:

• Emotional Power (EP): from EDA, this indicator reflects the strength of emotional reactions and is calculated using the amplitudes of the EDA phasic peaks in a given time frame. Studies have highlighted the specific link between the amplitude of SCRs and the sympathetic activation strength (Routtenberg, 1971; Lockhart, 1972; Pribram and McGuinness, 1975; Fowles, 1980; Gray, 1982; DeLong et al., 1983; Boucsein, 2012). Furthermore, the scientific literature has demonstrated the existence of several distinct activation systems (Routtenberg, 1971; Pribram and McGuinness, 1975; Fowles, 1980; Gray, 1982; DeLong et al., 1983; Boucsein, 2012): one of these systems is involved in affective activation or effort and is characterized by short phasic responses with a high amplitude approaching reflex responses and causing inhibition of subcortical structures involved in motivation and cognitive tasks, it is a fight/flight type response.
• Emotional Density (ED): from EDA, this indicator reflects the frequency of emotional reactions and is calculated using the number of EDA phasic peaks in a given time frame. An activated organism is characterized by a lowering of perceptual thresholds and an increase in the activity of central alert-related structures, resulting in bursts of sympathetic activity and thus SCRs. Following the lowering of sensory thresholds (confirmed by the increase in the activity of the somatomotor areas) and the need for reactivity (neuronal bursts of sympathetic activation), the frequency of occurrence of peaks increases, without affecting the amplitude, when the body is in a state of prolonged activation (Munro et al., 1987). This variation in the frequency of peak appearance, called lability, is related to emotional factors and the regulation of affect (Crider, 2008; Sarchiapone et al., 2018).
• CL: from EDA, this indicator reflects the amount of mental effort exerted and is calculated from the slow-moving tonic components of EDA in a given time frame. The tonic level is related to the general activation of the body, this indicator varies slowly over time. An increase in tonicity is observed when the situation leads to sustained engagement and attention (thus the opposite of a fight/flight type reaction, short and powerful; Boucsein, 2012; Saitis et al., 2018).

This approach takes into account the variation of objective measurements in various tasks already in use in the scientific community and the Affect-tag indicators are based only on those and not on subjective measurements. The creation of this database and the purpose of this testing is to further refine specific parameters and minimize errors for each Affect-tag indicator.

## Methods

### Participants

The study included 50 adults (25 males, 25 females) between the ages of 20 years and 55 years. All testing took place between May 2 and July 15, 2019. Recruitment was carried out in different locations in the north of France to diversify recruitment and all participants were paid 10€ for their involvement.

### Procedure

Testing took place at the Imaginarium in Tourcoing, France, and other local laboratories. Upon arrival, participants signed a consent form, the Affect-tag band was placed on the left wrist, and they were seated in front of a laptop computer. The participants completed the PANAS questionnaire to correct anxiety levels and their influence on physiological signals. All instructions and tasks were displayed using E-Prime without any operator intervention. A marker was used to align the physiological data acquisition from Affect-tag with the start of the E-Prime program and for each task.

The tasks were as follows:

• Reaction time task
• Breathing exercises
• Mental calculation
• A video sequence, with negative valence
• Several images, which may be pleasant or unpleasant
• Affective memory task
• Social stress test (preparation to sing out loud)
• A final calibration phase.

A 60-s decompression phase occurred between each task. Table 2 outlines the progression of the tasks.

**Table 2:** Progression of emotional and cognitive tasks tested.

Task	Time (s)	Purpose
Introduction	10	
Baseline 1	60	Calibration
Instruction: countdown and reaction	15	Reaction: decision and action taking
Reaction task	8
Baseline 2	60	
Instruction: breathing	20	Autonomic regulation
Breathing tasks	120
Baseline 3	60	
Instruction: mental workload	20	Cognitive load
Cognitive load (weak)	30
Cognitive load (strong)	60
Baseline 5	60	
Instruction: affect videos	10	Emotions and dtress
Emotional task: emotional stress	60
Instruction: images	10	Affects
Emotional task: images	390
Emotional task: affective memory	45
Baseline 6	60	
Instruction: social	10	Social stress
Emotional task: social stress	20
Baseline 7	5	

*The purpose of each phase and their lengths are noted. All participants followed a programmed paradigm with unvarying lengths*.

All data is anonymous and no participant information is retained except for age and gender.

### Stimuli

#### Affects

The International Affective Picture System (IAPS, Lang et al., 2008) database was used for the video and image sequences based on two factors: valence (positive and negative) and activation (low and high). Each block of images had a mean valence score and a mean arousal score. These values (along with their standard deviations) are presented in Table 3.

**Table 3:** Mean valence and arousal scores for the images taken from the International Affective Picture System (IAPS) used during the passive testing phases.

	Valence and arousal scores	
	**Low arousal**	**High arousal**
Positive valence	**6.5 (0.3)** *2.7 (0.1)*	**7.5 (0.2)** *7 (0.3)*
Negative valence	**2.9 (0.6)** *4 (0.3)*	**1.6 (0.2)** *7 (0.2)*

*Values in bold represent valence, values in italics represent arousal. The standard deviations are in parentheses*.

Five images were presented for each block for a total of 75 s per block. Between each block, two neutral images were presented to neutralize the emotion.

The affective memory task entailed showing images related to the video with negative valence shown just before. Three images, which included characters from the video, designed to evoke an emotional response were shown to participants to test the presence of affective memory.

#### Cognitive Load and Autonomic Regulation Tasks

The CL tasks required participants to perform 25 arithmetic operations and record the result for each on a response sheet provided. This test allows for variation in mental load level by adjusting the complexity of the operations. The first 10 operations are very simple to perform (e.g., 2 + 1 + 4) while the final 15 are more complex (e.g., 3–9 + 7).

The breathing tasks involved a short period of inhalation, holding the breath, and a long period of exhalation because this respiratory time ratio increases cardiac parasympathetic tone. This method was adapted from Strauss–Blasche et al. (2000).

## Analysis

### Data Preparation

Data from the first two participants were excluded due to procedure adjustments. Data for 24 males and 24 females were analyzed.

The precise timing for responses and execution of each task was imported from E-Prime to synchronize it with the physiological data from Affect-tag. Data for everyone was then stored into phases by task, with the image tasks being separated by each block and the mental calculation phases separated into “easy” and “difficult”. To remove the contamination of the previous block, the instruction phases were used as the baseline.

### Method Validation

To ensure the validity of each presented task, statistical significance was performed on the results. The following sections explain the validation method.

#### Anxiety Levels

The participants were asked to take the PANAS questionnaire to verify their capability to balance positive and negative affects. Analysis revealed that the distribution of scores on the positive scale is Gaussian and centered on a value slightly higher than the theoretical average. The distribution on the negative scale, however, is rectangular and centered on the minimum values of the scale. It can therefore be concluded that the population tested has rather positive feelings (mean = 27.39) without the intrusion of negative affects (mean = 13.06). This population, therefore, leans to a more positive affect.

#### Reaction Time

Analysis of the reaction times revealed that four participants did not complete the task as expected, the reaction times were too long for the type of task, indicating the response was not detected by the computer.

In this quite simple task (pressing a key on the keyboard), reaction times are usually very short (less than 2 s). But for four participants, this time was more than 4 s, which is very suspicious. We believe that the participants did not press the keyboard key hard enough for the computer to detect the response.

**Figure 2:**
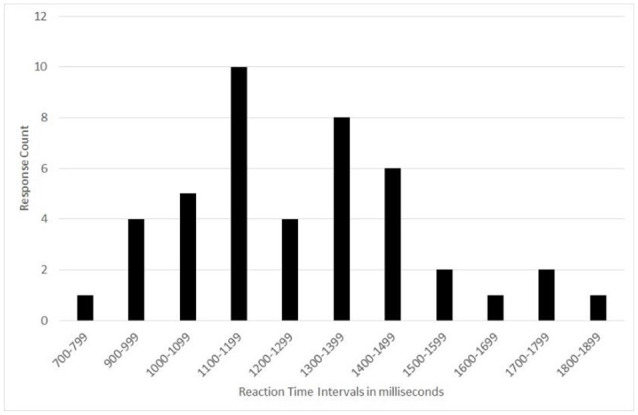
Number of participants vs. reaction time intervals during the reaction time tests showing an asymmetric Gaussian distribution.

After removing these four participants from the global analysis, the distribution of results is asymmetric Gaussian. Therefore, the median was used as the central index rather than the average (Figure 2).

#### Calculation Task

The 25 arithmetic operations were separated into weak and strong levels of mental load. The success rate of the arithmetic problems for the weak mental load (mean = 84%) was statistically significant and lower than the success rate of the strong mental load problems (mean = 61, *t*_(48)_ = −7.96, *p*-value = 0). The items chosen for this experiment, therefore, make it possible to create two very different levels of difficulty.

### Affect-tag Cogtech

The Affect-tag Cognitive Technology (CogTech) is first applied using the electronic components built into the wristband. The EDA is measured and transformed into readable data that are displayed in real-time on the Affect-tag mobile application.

For offline analysis, a “sanitation” algorithm was applied to the raw EDA signal to determine signal quality and reconstruct data identified as artifacts. This algorithm includes correction of gaps or duplicate data entries and an application of a median filter to reconstruct artifacts identified as noise or movement artifacts. A Signal Quality Index (SQI) score was calculated for each data set for the length of the signal (mean = 95%) and for each separated phase of the data. A threshold is set for an acceptable level of correction; if there are too many artifact corrections in the signal and the SQI is too low, that phase of the data will not be included in the analysis. For optimization of indicators, the threshold for acceptable signal quality was a variable that was tested.

Affect-tag tonic algorithms extract the tonic component of the measured EDA signal by using a series of specially designed signal filters. The Ledalab Continuous Decomposition Analysis (CDA) method was used as the gold standard for EDA signal decomposition (Benedek and Kaernbach, 2010; Brouwer et al., 2017) for the development and refinement of the Affect-tag algorithms. From the Ledalab documentation: “this method extracts the phasic (driver) information underlying EDA and aims at retrieving the signal characteristics of the underlying sudomotor nerve activity (SNA).” When correlated with the Ledalab CDA algorithms, the Affect-tag algorithms achieved a 98% correlation over 30 tests from data sets in multiple contexts. Before being displayed or recorded, an artifact detection algorithm is passed to remove artifacts in the tonic component based on signal shape and physiological feasibility.

The fast-changing phasic component peaks are detected using a set threshold level, time minimum between detected peaks, and achievable slope. Normalization is applied to both the tonic and phasic components to perform interindividual analysis and compare results across participants.

### Accuracy Metrics

Before calculating the Affect-tag emotional indicators for each phase of the test, the expected outcomes by indicator were defined. For example, we expect to see a significant positive difference between the reaction task and baseline for EP but the same comparison is not of interest for CL where a much longer time is needed to measure an effect. A summary of the expected outcomes for each task and the baseline to which they were compared is shown in Table 4.

**Table 4:** Comparisons by task: the phases used as the baseline are listed in the first column and the tasks are listed in the second column.

		EP		ED		CL	
Baseline	Task	Sig. Diff. Exp.	*P*-value	Sig. Diff. Exp.	*P*-value	Sig. Diff. Exp.	*P*-value
Baseline 1	Reaction task	X	<0.001	X	0.007		
Instruction: breathing	Breathing task	X	0.047		0.687		0.374
Baseline 3	Low cognitive charge		0.416	X	0.003	X	0.310
Baseline 3	High cognitive charge		0.125	X	<0.001	X	0.001
Low cognitive charge	High cognitive charge		0.292	X	0.027	X	0.003
Instruction: affect videos	Video task (negative valence)	X	0.174	X	0.037		0.769
Image: neutral	Image: low arousal	X	0.359	X	0.787		0.353
Image: neutral	Image: high arousal	X	0.363	X	0.904		0.028
Image: low arousal	Image: high arousal	X	0.777	X	0.725		0.047
Image: neutral	Affective memory task	X	0.131	X	0.138		0.060
Baseline 5	Social stress: instructions	X	<0.001	X	0.250		0.007
Baseline 5	Social stress: countdown	X	0.043	X	0.059		0.003
Baseline 5	Social stress: singing	X	<0.001		0.193		
Baseline 5	Social stress: all		<0.001		0.083	X	0.001

*Each line represents a different test case (baseline vs. task) where the Affect-tag Emotional Indicators were compared. The last three columns separated by Affect-tag indicator include an “X” where there was a significant difference expected for that indicator (EP, emotional power; ED, emotional density; CL, cognitive load). The columns in gray are the passive tasks that incorporated images or video with positive or negative valence. These results were considered inconclusive due to the affective tendancies of the population tested. It is to be noted that *t*_(46)_ = 2.013 for *p* = 0.05*.

Some phases, such as Baseline 2, were omitted from the analysis due to possible contamination with data from other testing windows. This decision was made after the testing procedure was carried out and to ensure the slow EDA changes have had ample time to return to baseline in between tasks.

To refine the algorithm that calculates the emotional indicators, certain parameters were altered to attain results that were congruent with hypotheses and significantly different. The following parameters were modified until an acceptable error emerged:

• SQI threshold
• Minimum peak detection threshold
• Amplitude correction
• Stimulus window
• Minimum acceptable time between successive peaks
• Normalization method

## Results

### Validity of Passive Tasks

The analysis of the PANAS results revealed that the population tested was not balanced between positive and negative affect. Results for emotional measurements could therefore be skewed, with values for EP and ED not reflecting the general population. The test phases were therefore separated into “active” tasks and “passive” tasks, with the passive tasks being those using the valence and arousal media and the active tasks being the phases where participants were invited to perform an action. Since the analysis revealed that the analysis of the passive tasks was not representative of the population, they have been ruled inconclusive.

### Statistical Significance Tests

A two-sided Student’s *t*-test (*t*_(46)_ = 2.013) was performed for every test case listed in Table 4. The associated mean differences in indicators’ values for each test case are listed in Table 5.

**Table 5:** Difference by task: the phases used as the baseline are listed in the first column and the tasks are listed in the second column.

Baseline	Task	ΔEP (%)	ΔED (%)	ΔCL (%)
Baseline 1	Reaction task	10.60	6.80	
Instruction: breathing	Breathing task	2.23	0.66	−1.25
Baseline 3	Low cognitive charge	1.28	5.02	0.66
Baseline 3	High cognitive charge	3.85	8.44	4.05
Low cognitive charge	High cognitive charge	2.57	3.42	3.39
Instruction: affect videos	Video task (negative valence)	−2.90	−5.48	−0.35
Image: neutral	Image: low arousal	−1.19	0.21	0.44
Image: neutral	Image: high arousal	−0.94	−0.10	−1.22
Image: low arousal	Image: high arousal	0.25	−0.31	−1.67
Image: neutral	Affective memory task	−2.63	2.02	−3.37
Baseline 5	Social stress: instructions	11.22	3.55	5.90
Baseline 5	Social stress: countdown	7.85	5.91	8.04
Baseline 5	Social stress: singing	25.40	4.09	
Baseline 5	Social stress: all	13.35	4.36	9.81

*Each line represents a different test case (baseline vs. task) where the Affect-tag Emotional Indicators were compared. The last three columns separated by Affect-tag indicator include the mean difference in value between the two phases compared (EP, emotional power; ED, emotional density; CL, cognitive load, all indicators are expressed in percentages so the difference in value is also expressed in percentages)*.

The Emotional Power indicator reached significant differences for the reaction time task, breathing task, and all social stress situations (*p*-value <0.05). There were no significant differences for the cognitive charge tasks or the arousal images; however, the results were consistent with expectations. The affective memory and negative valence tasks produced no effect.

The ED indicator reached significant differences for the reaction time task and cognitive charge tasks. There were no significant differences for the arousal images showed and social stress tasks but, however, the countdown part of the social stress tasks exhibited a trend towards significant (*p*-value = 0.059).

The CL indicator reached significant differences for the social stress tasks and comparisons using high cognitive charge (*p*-value <0.001). Only the comparison between baseline and low cognitive charge had a non-significant difference yet it exhibited a trend in the expected direction.

### Accuracy Representation

To go further, for every test case, an experimental score was assigned for each indicator based on the significance of the expected results. This permitted calculation of a weighted accuracy for each indicator in the active tasks for which they are essential. In Table 6 below are the results for each indicator.

**Table 6:** Accuracy for each Affect-tag Emotional Indicator based on test cases with statistically significant results.

	EP	ED	CL
# test cases with sig. diff. / number of test cases	5/5	5/6	5/6
Accuracy score	**100%**	**83%**	**83%**
Global Accuracy Score		**89%**	

## Discussion

To provide scientifically validated affective and cognitive indicators, it was important to create a database of expected physiological behavior. This database served as a basis for the refinement of the Affect-tag indicators which resulted in viable representations of emotional intensity, frequency, and CL.

The database for the affective tasks remains inconclusive. Affective memory depends on the ability to call on the memory of details from a similarly emotional situation. To have a database that can be generalized to the population, a normal distribution of affective responses is required on the PANAS questionnaire. An acceptable database can be obtained by measuring additional participants that result in a normal distribution of affective sentiments.

Perhaps a redesign of the paradigm to measure affect is required to ensure an accurate database. The IAPS, for example, was created for measuring certain pathologies vs. control groups. This database consists of only a control group with no specific pathology to measure against.

The hardware used in our study is a device that was entirely imagined and built by a start-up company. This required the construction of a wristband containing the sensors and embedded electronics, programming the firmware and the development of an application to control the device and conduct experiments using an adapted interface, to proceed with automated online data analyses, and finally to produce a final online report on a web application.

A native solution based on the recommendations of the Society for Psychophysiological Research Ad Hoc Committee on Electrodermal Measures (2012) was developed and, at this time, the deconvolution method proposed by Benedek and Kaernbach (2010) seemed to be the most promising one (Boucsein, 2012). However, new methods have recently appeared and are currently being studied for implementation in our systems. Although the results of the study are encouraging, the fact that only one type of algorithm for signal decomposition was tested in this study is a limitation. Further studies are therefore necessary in order to optimize signal processing using the latest techniques. For example, the convex optimization approach (cvxEDA, Greco et al., 2015) and the sparse deconvolution approach (sparsEDA, Hernando-Gallego et al., 2017) are relatively fast techniques that can be implemented in wearable devices for a lower computational cost. Moreover, the convex optimization approach can be better to address inter-and intra-subject variability for a specific subject or condition and sparseEDA is a solution that allows for the fully automated extraction of phasic component from large and small EDA (Posada-Quintero and Chon, 2020). More interestingly, a new method based on spectral analysis of the electrodermal signal has recently been proposed (Posada-Quintero et al., 2016). The authors propose a new measure (termed TVSymp), based on the low frequency power spectra of the EDA. They found that this measure is highly sensitive to cognitive and physical stress, even exhibiting a higher between-subject consistency than did other measures of EDA. These methods became more prominent and widely accepted after the development of the Affect-tag EDA analysis algorithms using CDA, but they have not yet been used for this application. However, these techniques will be implemented in Affect-Tag for HRV analysis and therefore, it will become possible to combine spectral analyses of heartbeat and EDA, which will ensure more consistency between measurements and will make it possible to study new phenomena acting on both of these two measures in different ways and thus, through the study of these multiple signals, further open up possible case studies and interpretations.

This study shows that the Affect-tag sensor can measure physiological states accurately enough to detect changes in responses caused by tasks involving cognitive, emotional, and social functions. With this type of mobile system, it is possible to study people’s reactions in everyday situations (for example, stress detection in daily life, Can et al., 2019) or professional situations (for example, to study construction workers’ perceived risk, Choi et al., 2019), and above all, with a scientific rigor comparable to that of laboratories. The quality of the measurement is not the only factor to be considered here, the quality of the interpretation of this measurement is also important. It is therefore essential to have at one’s disposal a reference database constructed from measurements carried out under standardized conditions.

## Data Availability Statement

The datasets generated for this study are available on request to the corresponding author.

## Ethics Statement

The studies involving human participants were reviewed and approved by Comité d’Éthique Lille—Université de Lille. The patients/participants provided their written informed consent to participate in this study.

## Author Contributions

LS developed the theory. LS conceived and planned the experiment with the support of HS. A research engineer was employed by SCAlab to carry out the experiment. HS directed the study, processed the experimental data, and performed the analysis. LV aided in the analysis and drafted the manuscript. OJ supervised the project. All authors discussed the results and contributed to the final manuscript. All authors contributed to the article and approved the submitted version.

## Conflict of Interest

HS, LV and OJ were employed by the company NEOTROPE. The remaining author declares that the research was conducted in the absence of any commercial or financial relationships that could be construed as a potential conflict of interest.
